# A comparison of chronic kidney risk among returnee Nepalese migrant workers in the countries of the Gulf and Malaysia and non-migrants in Nepal: a population-based cross-sectional study

**DOI:** 10.1186/s12882-026-04872-7

**Published:** 2026-03-18

**Authors:** Nirmal Aryal, Pramod R. Regmi, Arun Sedhain, Sankalpa Bhattarai, Radheshyam Krishna KC, Shravan Kumar Mishra, Ben Caplin, Neil Pearce, Edwin van Teijlingen

**Affiliations:** 1 Faculty of Health, Environment and Medical Sciences, Bournemouth University, Bournemouth Gateway Building, BH8 8GP Bournemouth, UK; 2 National Academy of Medical Sciences (NAMS), Kathmandu, Nepal; 3 Green Tara Nepal, Kathmandu, Nepal; 4 Migration Health Division, International Organization for Migration, Tripoli, Libya; 5 Provincial Public Health Laboratory, Madhesh Pradesh, Janakpurdham, Dhanusha, Nepal; 6 Department of Renal Medicine, University College London, London, UK; 7 Department of Epidemiology and Population Health, London School of Hygiene and Tropical Medicine, London, UK

**Keywords:** Kidney health, Migrants, Cross-sectional study, Nepal, Gulf countries, Malaysia

## Abstract

**Introduction:**

There have been increasing concerns about the possible high prevalence of chronic kidney disease (CKD) among returnee Nepalese migrant workers from the Gulf countries and Malaysia. This population-based cross-sectional survey primarily aimed to examine the prevalence of kidney health risks of Nepalese recent migrants compared to non-migrants from the same community.

**Methods:**

We conducted a cross-sectional survey of 1438 participants from Dhanusha district (718 recent migrants and 720 non-migrants (including historic migrants)). Recent migrants (all sexes) aged 18 years or above who had stayed for at least two years in six countries of the Gulf region or Malaysia in any occupation and had returned in the past 12 months were included. We used the core questionnaire and protocol for the Disadvantaged Populations eGFR Epidemiology Study (DEGREE) and added questions on migration.

**Results:**

All recent migrants were male and they were compared to male historic migrants, male non-migrants, and female non-migrants. Only 6 (0.4%) cases of eGFR < 60mL/min/1.73m^2^ were identified overall. The prevalences of eGFR < 60mL/min/1.73m^2^ were 0.4% (95% CI: 0.08 to 1.2), 0.5% (95% CI: 0.01 to 2.6), 1.2% (95% CI: 0.1 to 4.2%), and zero in male recent migrants, male historic migrants, male non-migrants, and female non-migrants, respectively. The prevalence of high proteinuria (> 30 mg/g) was lowest among male recent migrants at 7.7% (95% CI: 5.8 to 9.8) and highest among female non-migrants at 10.5% (95% CI: 7.5 to 14.3). In the adjusted multiple regression model, male recent migrants had a statistically nonsignificant, slightly reduced mean difference in eGFR of -0.8 mL/min/1.73m^2^ (95% CI: -3.6 to 2.0) compared to male non-migrants. A separate adjusted model among male recent migrants showed a strong association between reduced mean eGFR and older age, occupation as a security guard, current or past smokers, and ethnicity (Terai Dalits and Muslims).

**Conclusion:**

This study found a low prevalence of eGFR < 60mL/min/1.73m^2^ in Nepalese recent migrants. There were no associations of mean eGFR by migration status despite male migrants being exposed to risk factors for kidney disease. Nonetheless, this study indicated that specific sub-groups of Nepalese migrants mainly those working as security guards and from certain ethnicities could be at higher risk. These require further investigations.

**Supplementary information:**

The online version contains supplementary material available at 10.1186/s12882-026-04872-7.

## Background

In Nepal, nearly 5 million work permits have been issued since 2008 for employment-related international migration, primarily to the Gulf Co-operation Council (GCC) countries and Malaysia [1]. The Nepal 2021 Census reported that 23.4% of households have at least one family member living abroad [2]. According to the Nepal Labour Migration Report 2022, labour migrants were mostly males, low-skilled, based in GCC countries and Malaysia, and had sent over US$ 8.8 billion in remittances, accounting for one-quarter of the national income [1].

In recent years, concerns about kidney health risks among Nepalese labour migrants returning from GCC countries and Malaysia have sparked significant scientific discussion both nationally and internationally [3–9]. Although evidence is limited, some hospital-based studies suggest a possible higher prevalence of kidney problems among returnee migrant workers. A 2023 study conducted at two large haemodialysis treatment centres in Nepal found that 28% of kidney failure patients were returnee migrants, who also experienced kidney failure at a significantly younger mean age than non-migrant patients [10], though this could simply reflect the fact that migrants tend to be younger than non-migrants overall. A tertiary care centre in eastern Nepal also reported that 31% of patients on maintenance dialysis were returnee migrants [11]. More than two-thirds of 217 returnee Nepalese migrants who had a kidney transplant between 2013 and 2023 reported having worked in temperature over 40^0^ Celsius and 78.1% had a daily water intake of less than two litres [12]. An online survey of Nepal-based nephrologists found that more than two-thirds believed migrants face a higher kidney health risk than the general Nepalese population [3].

The recent qualitative study with 12 returnee Nepalese migrants from the GCC countries and Malaysia diagnosed with kidney-related problems identified poor access to potable water, excessive use of pain medication, poor diet, and occupational pollutants as potential risks [13]. A longitudinal study of 65 Indian construction workers in Saudi Arabia reported that 18% suffered from kidney injury, suggesting exposure to heat, long working hours, dehydration, sleep deprivation, and obesity as risk factors [14]. A 2021 Amnesty International report also documented that doctors in South Asia observed high levels of kidney health issues among returnee migrants from the GCC countries and Malaysia [8].

Nepal lacks a national disease surveillance system, and hospitals do not record data based on migration status. Consequently, the health conditions of returnee migrants are not documented unless they seek financial compensation for one of the 15 critical illnesses, including kidney failure. Regular health screening for migrant workers in GCC countries is not legally mandated and is often irregular, making it difficult to identify incremental health risks. As a result, it is challenging to determine whether the high number of returnee migrants with kidney failure or health issues seeking treatment in Nepal’s hospitals indicates a similar risk within the general population or if it signifies a disproportionately higher risk associated with their migration abroad.

A potential higher kidney health risk among Nepalese migrants should not be ignored as they are young (median age 28, with half between 25 and 34 years) [1]. Additionally, the working and living conditions of many migrant workers in GCC countries and Malaysia involve known risk factors for kidney health, such as exposure to extreme heat, heavy workload, dehydration, excessive use of alcohol, sugary drinks, and non-steroidal anti-inflammatory drugs (NSAIDS) [13, 15]. Evidence strongly suggests that migrating from low-income to high-income countries increases the risk of obesity and diabetes due to changes in dietary and lifestyle habits [16]. These factors, individually or combined, can impair kidney function.

Against this background, we hypothesised that returnee migrants may face higher kidney health risks compared to non-migrants. The main aim of this study is to produce the first population-based evidence on the prevalence of kidney health risks and associated risk factors among returnee migrant workers, and to compare these with non-migrant populations in Nepal within the district with the highest transnational outmigration.

## Methods

This was a survey of the source population with previous work-related migration history as the primary exposure of interest. Data were collected between 11 March and 26 May 2023 in two municipalities (Laximiniya and Chhireshowrnath) of Dhanusha district. Accordingly, recruitment focused on Laximiniya (rural) and Chhireshowrnath (urban) municipalities. Dhanusha district, in the east of the country, was selected as it has the highest number of labour out-migrants in Nepal [1], with the vast majority being low-skilled (e.g., construction and factory) workers. Potential participants were invited if they: (i) were recent migrants (who had returned in the past 12 months), aged 18 years and over of all sexes; had stayed at least two years (to allow for exposure to migrant worker experience) in GCC countries or Malaysia in any occupation, or (ii) were non-migrants or historic migrants (who had returned more than 12 months ago), aged 18–50 years of all sexes; and had lived in the study area for at least two years. All potential participants were required to speak and understand Nepali. For all groups, those unable to provide written or verbal consent, pregnant women, or individuals with severe impairments were excluded.

Considering the 5% prevalence of eGFR < 60mL/min/1.73m^2^ in the non-migrant population from the likely age group in Nepal [17] and assuming eGFR < 60mL/min/1.73m^2^ prevalence twice as likely as in the non-migrant population (10%), we estimated 435 samples in each migrant and non-migrant group (i.e., 870 in total) yielding 80% power and 5% margin of error [18]. While adjusting for a 1.5 design effect and a 10% non-response rate, we required 1,436 samples in total (718 per group).

### Sampling technique

A multistage sampling method was applied. As the sampling frame for migrants by administrative unit was unavailable, the study team consulted with relevant stakeholders and local authorities to identify the urban or rural municipalities in Dhanusha district with the highest number of out-migrants. In each municipality (Laximiniya and Chhireshowrnath), information on recent migrants was collected and approached to confirm eligibility criteria.

**Fig. 1:**
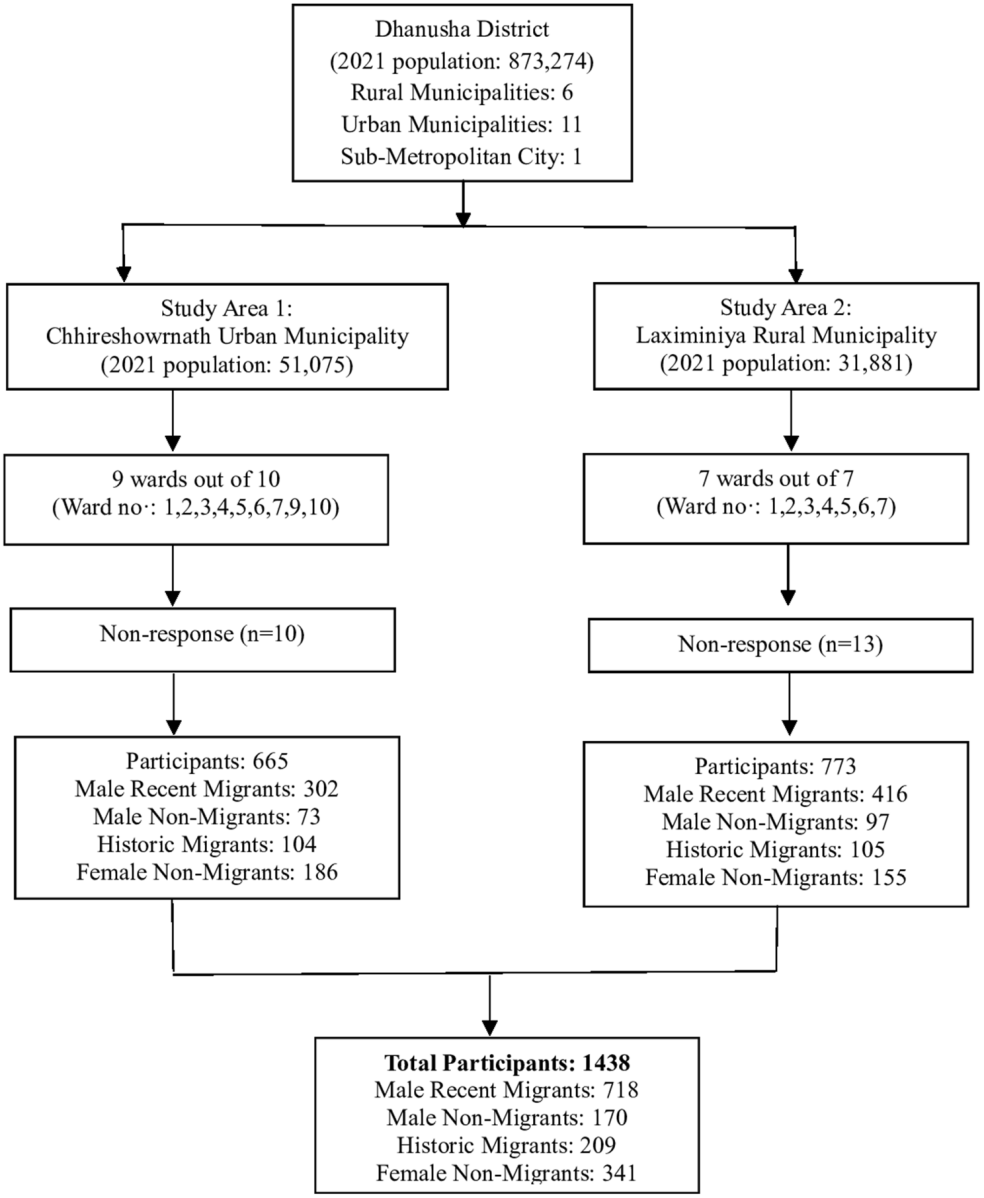
Study areas and sampling frame segregated by participant number and migration status

Those meeting the inclusion criteria from their households were invited. In households without migrants, only one participant meeting the inclusion criteria out of every seven households was invited. This was based on our mapping exercise which estimated that 12.5% of households in the district had at least one returnee migrant. The process continued until the required number of participants was recruited, totalling 16 wards (the lowest administrative units) from two municipalities. A study flow diagram detailing study areas and participants is presented in Fig. 1.

### Data collection tool and operational definitions

A survey tool was developed based on the core questionnaire for the Disadvantaged Populations eGFR Epidemiology Study (DEGREE) [19]. The questionnaire had six sections; socio-demographic variables (nine items), lifestyle and occupation-related variables (19 items), personal and family medical history (15 items), migration history and living and working condition-related variables while abroad (35 items, for recent migrants only), bio-physical measurements (blood pressure, height, weight), and kidney health-related laboratory tests (serum creatinine, blood sugar, uric acid, urinary albumin to creatinine ratio and seven items of urinalysis). The questionnaire was translated into Nepali and pre-tested with five returnee migrants to ensure clarity, intelligibility, and cultural sensitivity. Following the pre-testing, mainly questions on exposure to pesticides and occupation were amended to suit the local context and practice.

The eGFR was calculated using the 2021 CKD-EPI equation [20, 21]. A low eGFR suggestive of underlying CKDu was defined by excluding common known causes of CKD, i.e., the absence of diabetes mellitus, hypertension, or urinary evidence of glomerulonephritis in the form of heavy proteinuria [19, 22].

Sex was defined as assigned at birth. Blood pressure was classified as hypertensive if the mean systolic or diastolic blood pressure was equal to or greater than 140 mmHg or 90 mmHg, respectively, or if participants were on antihypertensive medication. Participants were classified as living with diabetes if their fasting or non-fasting blood glucose level was equal to or greater than 126 mg/dL or 200 mg/dL, respectively or if they were on antidiabetic medication. Proteinuria and heavy proteinuria were defined as high if the urinary albumin to creatinine ratio (ACR) was greater than 30 mg/g and equal to or greater than 300 mg/g, respectively. Overweight or obesity was defined by a body mass index (BMI) equal to or greater than 25 kg/m^2^.

### Data collection techniques

Two laboratory technicians and five research assistants (RAs) with public health/nursing degrees were employed. Data were collected in the morning as participants were likely to be at work during the day. If absent, up to three attempts were made before the next eligible participant on the list was approached. The RAs completed a paper version of the questionnaire. Laboratory technicians collected blood (5 mL) and urine samples at the same time. Blood samples were stored at 2–8^0^ C using a cold pack and transported to the laboratory the same day. We took a sample from each batch and recorded the batch variation. The project coordinator supervised field data collection, monitored data quality, and addressed any issues at the field level. A repeat test of serum creatinine and proteinuria was planned after 3 months for participants with eGFR < 90 ml/min/1.73m^2^ or urinary ACR greater than 30 mg/g. Serum creatinine assays were referenced according to the Isotope Dilution Mass Spectrometry (IDMS) standard.

With participants’ consent, the RAs reviewed medical records, if available, and recorded relevant information. These records were requested directly from the participants because there was no other way to access them.

Participants were provided with a participant information sheet detailing the study and what participation would involve. Written consent was obtained where possible; if participants were illiterate, witnessed consent was obtained. Participants with abnormal results were also referred to the nearest hospital with a nephrologist.

The STROBE statement was followed for reporting the results.

### Statistical analysis

Chi-squared and Analysis of Variance (ANOVA) tests (or its non-parametric counterpart Kruskal-Wallis) were used to compare migration categories with categorical (e.g., hypertension, diabetes) and continuous outcomes (e.g., eGFR, proteinuria), respectively. Multiple linear regressions were used to (i) estimate the association between mean eGFR and migration status, and (ii) identify factors associated with reduced eGFR among recent migrants adjusted with the potential confounding variable (age) and known risk factors. The selection of variables (both continuous and categorical) in multiple linear regression models was based on relevance to the research context. Assumptions for the linear regressions were checked and found to be reasonably met. Stata software (Version 17) was used for analysis. Among historic migrants, only one was female and thus excluded from data analysis for male-to-male comparisons.

We could not access a significant number of eligible participants during the repeat tests after three months, particularly for recent migrants (45.2% for reduced eGFR and 65.4% for high proteinuria were missing), and hence only the results of the first blood and urine tests were included here.

### Patient and public involvement and engagement (PPIE)

We held consultation meetings with key stakeholders working for migrant communities, researchers, health care providers and migrant workers in Malaysia and Nepal to ensure that the issue of kidney health risks among Nepalese migrants is significant and warrants further investigation [23]. Members of the research advisory group, including returnee migrants and organisations focused on migration health in Nepal, contributed to formulating research questions, exploring various study designs considering given time and resource constraints, translating study tools, piloting the study questionnaire, and disseminating study findings.

## Results

Twenty-three potential participants (1.6%) refused to take part, citing reasons such as fear of needles and possible effects on their migration plans. A total of 718 male recent migrants, 209 male historic migrants, 170 male non-migrants, and 342 female non-migrants participated. The 209 male historic migrants had mainly worked in the six Gulf countries and Malaysia (94.7%) but had returned at least one year earlier.

Key socio-demographic characteristics, lifestyle risk factors, biological risk factors, and medical history of the study participants are shown in Table 1. The mean age of both male recent migrants (mean 35.0 years, SD 8.7) and male historic migrants (mean 35.0 years, SD 5.8) was the same, which was significantly higher than that of other groups. Around two-thirds of male recent migrants, male historic migrants, male non-migrants, and more than half of the female non-migrants were from similar ethnic groups (‘castes’). Male recent migrants reported a higher monthly mean income than the other groups (including all sources and from family members sharing the same kitchen), and the mean difference among the groups was statistically significant.

**Table 1:** Key socio-demographic characteristics, lifestyle, biological risk factors, and medical history

Variables	Male recent migrants *n* = 718 (six Gulf countries = 602, Malaysia = 116)	Male historic migrants *n* = 208	Male non-migrants *n* = 170	Female non-migrants *n* = 342
**n (%)**				
**Socio-demographic characteristics **				
Age (years)				
20–29	233 (32.4)	46 (22.1)	119 (70.0)	110 (32.3)
30–39	266 (37.0)	105 (50.5)	30 (17.6)	179 (52.3)
40–49	163 (22.7)	57 (27.4)	20 (11.8)	53 (15.5)
50 or over	56 (7.8)	0 (0)	1 (0.6)	0 (0)
Locality				
Urban	295 (41.1)	105 (50.5)	72 (42.3)	185 (54.1)
Rural	423 (58.9)	103 (49.5)	98 (57.6)	157 (45.9)
Ethnicity				
Terai other	440 (61.3)	152 (73.1)	120 (70.6)	180 (52.6)
Terai Dalit	131 (18.2)	29 (13.9)	25 (14.7)	104 (30.4)
Muslim	88 (12.3)	11 (5.3)	10 (5.9)	49 (14.3)
Terai Brahmin/ Chhetri	37 (5.1)	8 (3.8)	12 (7.1)	7 (2.0)
Other*	22 (3.1)	8 (3.8)	3 (1.2)	2 (0.6)
Education				
Illiterate	87 (12.1)	19 (9.1)	12 (7.1)	166 (48.5)
Informal education	48 (6.7)	1 (0.5)	3 (1.8)	36 (10.5)
Primary	246 (34.3)	64 (30.8)	22 (12.9)	52 (15.2)
Secondary	290 (40.4)	105 (50.5)	52 (30.6)	59 (17.2)
Higher Secondary	36 (5.0)	15 (7.2)	61 (35.9)	19 (5.6)
Bachelor’s or above	11 (1.5)	4 (1.9)	20 (11.7)	10 (2.9)
Occupation (except male recent migrants)				
Daily wage labour		45 (21.6)	30 (17.6)	39 (11.4)
Agriculture		68 (32.7)	29 (17.1)	26 (7.6)
Student		4 (1.9)	38 (22.3	7 (2.0)
Private sector employee		13 (6.2)	23 (13.5)	9 (2.6)
Self-employed		62 (29.8)	26 (15.3)	12 (3.5)
Homemaker		0 (0)	0 (0)	242 (70.8)
Other**		16 (7.7)	24 (14.1)	7 (2.0)
**Lifestyle risk factors**				
Smoked tobacco				
Current smoker	149 (20.7)	35 (16.8)	42 (24.7)	2 (0.6)
Never smoker	491 (68.4)	152 (73.1	113 (66.5)	338 (98.8)
Past smoker	78 (10.9)	21 (10.1)	15 (8.8)	2 (0.6)
Non-smoked tobacco				
Current user	491 (68.4)	156 (75.0)	98 (57.6)	1 (0.3)
Never user	184 (25.6)	42 (20.2)	68 (40.0)	341 (99.7)
Past user	43 (5.9)	10 (4.8)	4 (2.3)	0 (0)
Alcohol intake				
Current drinker	478 (66.6)	138 (66.3)	121 (71.2)	4 (1.2)
Never drinker	164 (22.8)	39 (18.7)	40 (23.5)	335 (97.9)
Past drinker	76 (10.6)	31 (14.9)	9 (5.3)	3 (0.9)
Physically heavy tasks at work	253 (35.2)	84 (40.4)	52 (30.6)	173 (50.6)
Exposure to pesticides	189 (26.3)	90 (43.3)	72 (42.3)	56 (16.4)
Addictive drugs use	26 (3.6)	8 (3.9)	12 (7.1)	0 (0)
**Biological risk factors and medical history**				
Hypertension or on medication	273 (38.0)	63 (30.3)	37 (21.8)	40 (11.7)
Diabetes or on medication	53 (7.4)	11 (5.3)	10 (5.9)	13 (3.8)
Overweight or obesity (≥ 25 kg/m^2^)	339 (47.2)	87 (41.8)	36 (21.2)	106 (30.9)
History of hypertension	64 (8.9)	18 (8.6)	7 (4.1)	21 (6.1)
History of diabetes	37 (5.1)	6 (2.9)	5 (2.9)	8 (2.3)
History of kidney disease	16 (2.2)	7 (3.4)	19 (5.6)	19 (5.6)
Family history of kidney disease	23 (3.2) (*n* = 717)	11 (5.3)	12 (7.1)	14 (4.1) (*n* = 341)
Ever use of traditional/herbal medicine	54 (7.5)	26 (12.5)	14 (8.2)	33 (9.6)
Snakebite ever	42 (5.8)	17 (8.2)	21 (12.4) (*n* = 169)	38 (11.1)
**Mean (SD)**				
Age (years)	35.0 (8.7)	35.0 (5.7)	27.8 (7.5)	32.8 (6.6)
Monthly household income (NPR)***	44,757 (39,405)	28,831(18,755)	42,181 (35,518)	36, 214 (25,066)
Meat intake days in a month	8.4 (7.1)	8.1 (7.2)	7.4 (6.6)	4.1 (4.1)
Exposure to pesticides (years) (*n* = 404)	7.7 (6.3)	7.1 (4.9)	6.1 (4.6)	5.6 (3.1)
Duration of work abroad (years)	10.1 (5.5)	6.0 (4.1)	-	-

*Ethnicity other: Terai Janajati,* Hilly Brahmin Chhetri*,* Hilly Janajati*

**Occupation other: Government employees,* homemaker male*,* unemployed*,* unable to work*

***1 GBP = Nepalese Rupees 169.15 (as of Jan 22,* 2025)*

*Responses from male recent migrants for lifestyle risk factors were related to Nepal rather than while working abroad.*

Non-migrant males had a higher prevalence of current smoking and current alcohol intake compared to other groups, and male historic migrants were more likely to be current non-smoked tobacco users. The differences in proportions for these variables were statistically significant. The most popular smokeless tobacco used by all groups was chewing plain tobacco leaves or mixed with betel nuts, and beer was the most common alcohol consumed. The prevalence of these risk factors among female non-migrants was negligible. Additional File 1 shows the bivariate analyses of the variables included in Table 1.

Male recent migrants worked in the GCC countries and Malaysia; more than half had worked in Qatar (Additional File 2), had worked abroad for an average of about ten years, and 54.6% were repeat migrants. More than two-thirds were involved in factory and construction work (Additional File 3).

Half of the male recent migrants were allowed just once or no rest time during the day, and on average took 2.4 (SD 0.1) days off per month (Additional File 4). One-quarter of jobs involved physically heavy work, and nearly half (45.5%) reported high heat exposure at work. More than one-third (38.3%) were always exposed to dust at work. Almost 10% were constantly exposed to chemicals, and just 1.8% were regularly exposed to pesticides. Very few migrants used painkillers regularly. The overwhelming majority reported easy access to water and toilets at work. Half of the migrants (53.5%) reported drinking alcohol while abroad, and around 40% drank one standard drink of alcohol at least 2–3 times per week. Beer was the most common type of alcohol for 80% of migrants, and 15.3% also drank counterfeit or home-brewed alcohol.

Compared to other groups, a higher proportion of male recent migrants were hypertensive (or on medication), diabetic (or on medication), and overweight or obese. After adjustment for age, male recent migrants and male historic migrants had significantly higher odds for overweight or obesity [Odds Ratio (OR) for male recent migrants: 2.15, 95% CI: 1.49 to 3.09; OR for male historic migrants: 1.56, 95% CI: 1.00 to 2.41] compared with male non-migrants. No such associations were observed for hypertension (or on medication) and diabetes (or on medication) after adjusting for age.

Overall, 49 participants (3.4%) had a history of kidney disease, which was significantly higher in male and female non-migrants (5.6% in each group) compared to male recent and historic migrants. Of these, the vast majority had kidney stones (*n* = 38, 77.5%), particularly among non-migrant females (*n* = 15, 30.6%) and male recent migrants (*n* = 12, 24.5%). A total of 60 participants (4.2%, *n* = 1436) reported having a family history of kidney disease, with a significantly higher proportion among non-migrant males compared to other groups. A minority of participants (8.8%) had ever used traditional/herbal medicine, mainly for gastritis issues, and this use was notably higher for male historic migrants.

Table 2 presents the prevalence of reduced eGFR and high proteinuria, as well as the mean values of these variables by migration status. Six participants (0.4%) (95% CI: 0.1 to 0.9) had eGFR < 60 mL/min/1.73m^2^ with no statistically significant difference in proportions between participant groups (*P* = 0.28). The eGFR < 60mL/min/1.73m^2^ prevalence was 0.4% (95% CI: 0.08 to 1.2) among male recent migrants, 0.5% (95% CI: 0.01 to 2.6) among male historic migrants, highest among male non-migrants (1.2%) (95% CI: 0.1 to 4.2), and zero among female non-migrants. Of these, two-thirds (4 out of 6, 66.7%) were from the age group 30 to 39 years. The age-adjusted prevalence of eGFR < 60mL/min/1.73m^2^ was 0.2% for male recent migrants, 0.2% for male historic migrants, and 1.2% for male non-migrants. Male recent migrants had the lowest proportion of high proteinuria (> 30 mg/g), and female non-migrants had the highest, but this difference was not statistically significant.

**Table 2:** Prevalence of reduced eGFR and high proteinuria and related mean values by migration status

Variables	Male recent migrants *n* = 718	Male historic migrants *n* = 208	Male non-migrants *n* = 170	Non-migrant females *n* = 342
**n** (%) (**95**% **CI**)				
*eGFR < 60* mL/min/1.73m^2^	*3 *(0.4) *(0.08 to 1.2)*	1 (0.5) *(0.01 to 2.6)*	2 (1.2) *(0.1 to 4.2)*	0 (0)
eGFR < 60 mL/min/1.73m^2^ without hypertension, diabetes, heavy proteinuria*	*0 (0)*	*0 (0)*	1 (0.6) *(0.01 to 3.2)*	*0 *(0)
eGFR < 90 mL/min/1.73m^2^	42 (5.8) *(4.2 to 7.8)*	11 (5.3) *(2.6 to 9.2)*	6 (3.5) *(1.3 to 7.5)*	9 (2.6) *(1.2 to 4.9)*
eGFR < 90 mL/min/1.73m^2^ without hypertension, diabetes, heavy proteinuria*	13 (1.8) *(3.2 to 9.9)*	6 (2.9) *(1.1 to 6.1)*	5 (2.9) *(35.9 to 99.6)*	8 (2.3) *(0.9 to 6.7)*
Proteinuria > 30 mg/g	55 (7.7) *(5.8 to 9.8)*	20 (9.6) *(5.9 to 14.4)*	15 (8.8) *(5.0 to 14.1)*	36 (10.5) *(7.5 to 14.3)*
**Mean (SD) (95% CI)**				
eGFR (mL/min/1.73m^2^)	112.8 (14.0) *(111.8 to 113.8)*	114.0 (12.6) *(112.3 to 115.7)*	119.7 (14.5) (*117.5 to 121.9)*	122.0 (11.2) (*120.8 to 123.2)*
Albumin to Creatinine ratio (mg/g)	13.2 (51.4) *(9.4 to 17.0)*	21.7 (97.9) *(8.4 to 35.3)*	27.8 (214.1) *(-4.6 to 60.2)*	17.3 (57.8) *(11.2 to 23.5)*
Serum creatinine (mg/dL)	0.9 (0.3) *(0.85 to 0.89)*	0.8 (0.1) *(0.83 to 0.87)*	0.8 (0.2) *(0.82 to 0.88)*	0.6 (0.1) *(0.57 to 0.59)*

*Heavy proteinuria: Albumin to Creatinine ratio ≥ 300 mg/g

Multiple linear regression estimated the association between mean eGFR and migration status adjusted for potential confounding variables and known risk factors (Table 3). In the adjusted model, the mean difference (95% CI) in eGFR among male recent migrants compared to male non-migrants was − 0.8 mL/min/1.73m^2^ (-3.6 to 2.0), with a wider confidence interval (*P* = 0.58). Female non-migrants had a significantly higher mean eGFR difference (95% CI) than male non-migrants; 4.1 mL/min/1.73m^2^ (1.2 to 7.1), *P* = 0.006. Higher age, history of kidney disease, current or past smokers and ethnicity were associated with the lower eGFR. Among ethnic groups, the Terai Dalit group had a significantly lower mean eGFR than the reference group, the Terai other. Sensitivity analyses were performed excluding outlier observations for eGFR, age, and BMI, which yielded a similar result, except for the negative association between current or past drinkers and mean eGFR.

**Table 3:** Multiple regression model for the association between mean eGFR and migration status adjusted for potential confounding variables and known risk factors

Variables	Estimate coefficient (95% CI)	*P* value
**Migration status** (ref: male non-migrants)		
Male recent migrants	-0.8 (-3.6 to 2.0)	0.58
Male historic migrants	0.05 (-2.9 to 2.9)	0.97
Female non-migrants	4.1 (1.2 to 7.1)	0.006**
Age (per decade older)	-8.6 ( -9.5 to -7.7)	< 0.001***
Yearly household income (per 50,000 Nepalese Rupees higher)	-0.01 (-0.09 to 0.06)	0.74
Meat intake days (per month)	-0.1 (-0.2 to 0.0008)	0.05
History of kidney disease (yes)	-6.7 (-11.8 to -1.50)	0.01*
Hypertension or diabetes or on medication for these (yes)	-0.9 (-2.5 to 0.7)	0.27
Current or past drinker (yes)	-1.3 (-3.2 to 0.6)	0.17
Current or past smoker (yes)	-2.4 (-4.0 to -0.7)	0.005**
BMI (per kg/m^2^ higher)	-0.1 (-0.3 to 0.03	0.12
History of snakebite (yes)	-0.7 (-3.2 to 1.7)	0.57
Exposure to pesticides (yes)	0.08 (-1.4 to 1.6)	0.92
**Completed education** (ref: Illiterate or informal education)		
Primary	0.02 (-1.8 to 1.8)	0.98
Secondary	-1.2 (-3.2 to 0.6)	0.19
Higher secondary or above	-2.2 (-5.2 to 0.7)	0.13
**Ethnicity** (ref: Terai other)		
Terai Janajati	-2.6 (-6.1 to 1.0)	0.16
Terai Dalit	-3.4 (-5.1 to -1.7)	< 0.001***
Terai Brahmin/Chhetri	-3.3 (-7.0 to 0.4)	0.08
Muslim	-2.0 (-4.0 to -0.004)	0.05
Other	-3.0 (-9.6 to 3.5)	0.36

P value: * (< 0.05), ** (< 0.01), *** (< 0.001)

Table 4 presents the multiple linear regression among male recent migrants, identifying the variables associated with reduced eGFR. Higher age, occupation as a security guard, being from the Terai Dalit and Muslim ethnic groups, and being current or past smokers were independently associated with reduced eGFR. Work-related circumstances speculated to pose kidney health risks showed a negative relationship with eGFR; however, the differences in risk were not statistically significant. No notable differences were observed during the sensitivity analysis that excluded outlier observations for age, BMI, eGFR, and migration years. Another multiple regression analysis excluding occupational mediators from this model yielded similar results except statistically significant negative associations between BMI and eGFR (Additional File 5).

**Table 4:** Factors associated with the reduced mean eGFR among male recent migrants adjusted with potential confounding variables and known risk factors

Variables	Estimate coefficient (95% CI)	*P* value
Migration duration (years)	0.02 (-0.2 to 0.2)	0.87
Age (per decade older)	-7.3 (-8.7 to -5.8)	< 0.001***
Yearly household income (per 50,000 Nepalese Rupees higher)	0.02 (-0.1 to 0.1)	0.65
Number of days off in a month	0.01 (-0.5 to 0.5)	0.95
Meat intake days (per month)	-0.04 (-0.2 to 0.1)	0.52
**Occupation** (ref: other^#^)		
Construction work	-2.7 (-5.7 to 0.1)	0.06
Factory work	-1.4 (-4.0 to 1.3)	0.31
Driving	-1.4 (-4.9 to 2.2)	0.45
Security guard	-9.0 (-15.1 to -3.0)	0.003**
**Physical intensity at work** *(ref: none or low)*		
Moderate	-0.05 (-2.8 to 2.7)	0.97
High	-0.7 (-3.2 to 1.9)	0.59
**Heat exposure at work** *(ref: none or low)*		
Moderate	-0.5 (-3.5 to 2.4)	0.72
High	1.1 (-2.1 to 4.3)	0.49
**Work setting** *(ref: indoor)*		
Indoor and indoor mix	-0.8 (-3.2 to 1.6)	0.49
Outdoor	0.6 (-1.8 to 3.1)	0.60
**Dust exposure at work** *(always or sometimes compared to never or rarely)*	-0.6 (-3.2 to 1.9)	0.64
History of kidney disease (yes)	-9.0 (-20.1 to 2.0)	0.11
Hypertension or diabetes or on medication (yes)	-1.6 (-3.9 to 0.8)	0.19
Current or past drinker (yes)	-1.4 (-4.0 to 1.2)	0.29
Current or past smoker (yes)	-2.3 (-4.3 to -0.3)	0.02*
BMI (per kg/m^2^ higher)	-0.2 (-0.5 to 0.01)	0.06
**Completed education** (ref: Illiterate or informal education)		
Primary	0.3 (-2.6 to 3.3)	0.82
Secondary	-0.7 (-3.5 to 2.2)	0.65
Higher secondary or above	-3.4 (-10.3 to 3.5)	0.33
**Ethnicity **(ref: Terai other)		
Terai Janajati	-1.5 (-5.7 to 2.7)	0.49
Terai Dalit	-5.3 (-7.9 to -2.6)	< 0.001***
Terai Brahmin/Chhetri	0.4 (-3.4 to 4.3)	0.83
Muslim	-3.1 (-6.1 to -0.1)	0.04*

P value: * (< 0.05), ** (< 0.01), *** (< 0.001); Other^*#*^*= mainly office work*,* sales*,* hotel work*,* cleaning*

## Discussion

This study found only 6 cases (0.4%) with eGFR < 60mL/min/1.73m^2^. The crude and age-adjusted prevalence of eGFR < 60mL/min/1.73m^2^ was higher among male non-migrants than among male recent migrants, male historic migrants, and female non-migrants. Two-thirds of these cases were reported among the younger age group (ages 30–39 years). Just one participant (male non-migrant) had eGFR < 60mL/min/1.73m^2^ in the absence of diabetes, hypertension or heavy proteinuria.

In the adjusted model, male recent migrants had a lower mean eGFR than male non-migrants; however, this difference was clinically insignificant, with a wide confidence interval. Male historic migrants and female non-migrants had higher mean eGFR than male non-migrants; this difference was statistically significant among female non-migrants only. A separate multiple regression among a subgroup of male recent migrants showed a strong association between a lower mean eGFR and occupation as a security guard.

The findings of the present study are similar to those of studies comparing Ghanaians in Europe and Ghanaians in Ghana, which showed higher cardiometabolic risk but lower CKD incidence and prevalence among migrants [24, 25]. The only nationally representative study in Nepal reported a prevalence of CKD among 4.4% of people aged 20 to 59 years old [26]; however, it used a different equation than ours for calculating eGFR and defined CKD as eGFR < 60 mL/min/1.73m^2^ and/or albumin to creatinine ratio of ≥ 30 mg/g. When compared to this national study and using the same criteria, our study reported a prevalence of 8.8% among all participant groups. The difference of at least five years in data collection points between the national study and the present study may indicate that the burden of kidney health problems is increasing rapidly at the community level. Systematic reviews targeting the general population of South Asia reported a higher prevalence of CKD than the present findings. Shrestha et al. [27] reported a pooled CKD prevalence of 15% in adult South Asian males, whereas Hasan et al. [28] reported a prevalence ranging from 10.6% to 23.3% across both sexes.

In the present study, an association between reduced mean eGFR and occupation as a security guard of recent migrants is of note. Security guards reported poorer work-related circumstances than those in other occupations; these included the longest daily working hours, the shortest daily rest time, the fewest days off per month, and the highest proportion with poor access to toilets. We could not identify any previously published quantitative evidence on these work-related conditions; however, our previous engagement with Nepalese migrant workers in Malaysia identified these issues among Nepalese security guards [23].

As expected, work-related circumstances of male recent migrants, such as high heat exposure, outdoor work, and physically heavy work, were associated with a quantitatively lower eGFR, but the associations were statistically nonsignificant.

The present study found a significantly higher prevalence of hypertension, diabetes, and overweight/obesity in male recent migrants and male historic migrants compared to male and female non-migrants. Compared to male recent migrants and male historic migrants, the latest World Health Organization (WHO) STEPS survey 2019 reported lower prevalences of these risk factors in the general adult population in Nepal aged between 15 and 69 years old, whilst these were similar to non-migrant males and females in the present study [29]. Interestingly, risk factors such as alcohol intake and smoking habits were more prevalent in non-migrant males. However, 15.3% of male recent migrants reported consuming potentially hazardous counterfeit or home-brewed alcohol while working abroad. Thus, both poor lifestyle habits and work-related circumstances may have contributed to cardiometabolic risks in male recent migrants while abroad.

The study also reported an independent association between reduced eGFR and participants from Terai Dalit populations, in separate adjusted models for all participants and male recent migrants only. Additionally, in a model including only male recent migrants, a statistically significant association was found between reduced eGFR and Terai Muslim participants. The Terai Dalit and Terai Muslim populations are socially and economically some of Nepal’s most disadvantaged and underserved population groups. Despite legal protections, they both face exclusion from state authority and economic resources. Dalits are mainly affected by caste-based “untouchability,” and Muslims are impacted by their religious identity [30]. A nationwide study also documented an association between Terai Dalit ethnicity and eGFR < 60mL/min/1.73m^2^ [26]. In the present study, participants from these ethnicities usually held physically demanding jobs; non-migrants from these groups were mainly daily wage labourers, and recent migrants were security guards and construction workers. High proteinuria was observed less commonly among male recent migrants than among other groups, whereas it was more prevalent among female non-migrants. A further investigation is warranted to ascertain the causes of high proteinuria in the young female population.

Despite the absence of a high burden of kidney disease, this study has further underpinned the precarious working conditions of Nepalese migrants in GCC countries and Malaysia (such as long working hours with one or no break time, unavailability of indoor rest place during the break, not taking weekly days off regularly, and regular exposure to heat) which could have negative health impacts [15].

### Strengths and limitations

This is the first population-based study worldwide to compare kidney health risks among migrants (from GCC countries and Malaysia) and non-migrants from the same community. This is also the largest study to date on Nepalese migrants, documenting work-related and lifestyle risks while working abroad. The main limitation is that we did not include the results of the repeat test (after three months) due to the low turnout rate. We could not access enough eligible participants during the repeat tests, particularly male recent migrants, because they usually have two months of leave every two to three years and have already gone abroad again for work. As a result, we did not meet the standard definition of CKD. The lack of female recent migrants has limited our study results to male migrants only and prevented us from comparing female migrants with female non-migrants. Female outmigration for employment is very low in the study district, which was the main reason for not being able to recruit female migrants. In 2023/24, just 251 females from Dhanusha district migrated compared to 19,942 males [31]. We selected study areas purposively to achieve the required sample size of migrants. Although a sample of migrants is random, a random sample of the whole population was not possible due to logistical and time constraints for recruiting enough migrants. We combined the data of recent migrants from the GCC countries and Malaysia for analysis, as the number of recent migrant samples from Malaysia was low. GCC countries and Malaysia have different working, living and environmental conditions for migrants. Finally, we do not know whether the CKD-EPI creatinine equation accurately estimates actual eGFR in Nepalese populations. Although this might affect the absolute number in the eGFR < 60mL/min/1.73m^2^ group, it does not affect the conclusion regarding differences between the groups.

The key policy recommendation for migration health policy planners in Nepal is to implement health screening for returnee migrants and to develop an integrated health database for migrants before and after their return. For employers and policy planners in destination countries, we suggest conducting regular workplace health camps, including simple anthropometric measurements and laboratory tests for cardiometabolic and kidney health risk markers, to identify potential risks earlier and prevent health deterioration. This is not only a moral obligation but also a strategy to enhance productivity. Regarding public health practice, the study findings underscore the need to promote health and raise awareness about lifestyle choices among Nepalese migrants in destination countries, as they were found to have a high prevalence of cardiometabolic risk factors such as hypertension, diabetes, and overweight or obesity. Nepalese embassies, local migration-related organisations, and public health practitioners should collaborate on this initiative.

In conclusion, this population-based study has identified a low prevalence of eGFR < 60 mL/min/1.73 m² in both migrant and non-migrant populations in Nepal. High levels of proteinuria were also less common among male recent migrants than in other groups. Notably, male recent migrants were not at a greater risk compared to non-migrant males, despite their exposure to risky work conditions and lifestyle factors. However, specific sub-groups of Nepalese migrants, particularly security guards and those from the Terai Dalit and Muslim ethnicities, may face higher kidney health risks that require further investigation. A longitudinal study of migrants that collects data periodically before their first departure, at destination countries, or after their return would provide more robust evidence on kidney health risks.

## Supplementary Information

Below is the link to the electronic supplementary material.

Supplementary Material 1: Bivariate analyses of key socio-demographic characteristics, lifestyle, biological risk factors, and medical history of participants categorised into four groups (male recent migrants, male historic migrants, male non-migrants, and female non-migrants)

Supplementary Material 2: Destination countries of current migrants (N=718)

Supplementary Material 3: Occupation of recent migrants during their recent employment

Supplementary Material 4: Working conditions and lifestyle characteristics of male recent migrants (N=718)

Supplementary Material 5: Factors associated with reduced mean eGFR among male recent migrants adjusted for confounding factors, known risk factors and excluding occupational mediators

## Data Availability

The dataset of this study is available from the corresponding author upon reasonable request.
