# Supplementary material for: A comparison of chronic kidney risk among returnee Nepalese migrant workers in the countries of the Gulf and Malaysia and non-migrants in Nepal: a population-based cross-sectional study
Source: BMC Nephrol. 2026 Mar 18;27:263. doi: 10.1186/s12882-026-04872-7 (PMC13122860; doi:10.1186/s12882-026-04872-7)
Supplement: Supplementary file 1 — Supplementary Material 1: Bivariate analyses of key socio-demographic characteristics, lifestyle, biological risk factors, and medical history of participants categorised into four groups (male recent migrants, male historic migrants, male non-migrants, and female non-migrants) [file 12882_2026_4872_MOESM1_ESM.docx]

**Additional File 1: Bivariate analyses of key socio-demographic characteristics, lifestyle, biological risk factors, and medical history of participants categorised into four groups (male recent migrants, male historic migrants, male non-migrants, and female non-migrants)**

*1. Categorical variables (Chi-squared test)*

A· Socio-demographic characteristics

(i) Age-groups: Pearson X^2^ (9, n=1438)=182·4, P <0·001

(ii) Locality: Pearson X^2^ (3, n=1438) = 18·5, P <0·001

(iii) Ethnicity: Pearson X^2^ (18, n=1438) = 519·7, P <0·001

B· Lifestyle risk factors

(i) Smoked tobacco: Pearson X^2^ (6, n=1438)=132·7, P <0·001

(ii) Non-smoked tobacco: Pearson X^2^ (6, n=1438)=580·7, P <0·001

(iii) Alcohol intake: Pearson X^2^ (6, n=1438)=634·7, P <0·001

(iv) Physically heavy tasks at work: Pearson X^2^ (3, n=1438)=28·8, P <0·001

(v) Exposure to pesticides: Pearson X^2^ (3, n=1438)=64·9, P <0·001

(vi) Addictive drugs use: Pearson X^2^ (9, n=1436)=56·7, P <0·001

C· Biological risk factors and medical history

(i) Hypertension or on medication: Pearson X^2^ (3, n=1438)=83·0, P <0·001

(ii) Diabetes or on medication: Pearson X^2^ (3, n=1438)=5·5, P=0·14

(iii) Overweight or obesity: Pearson X^2^ (3, n=1438)=52·6, P <0·001

(iv) History of hypertension: Pearson X^2^ (3, n=1438)=6·0, P=0·11

(v) History of kidney disease: Pearson X^2^ (6, n=1438)=81·7, P <0·001

(vi) Family history of kidney disease: Pearson X^2^ (6, n=1436)=90·5, P <0·001

(vii) Ever use of traditional/herbal medicine: Pearson X^2^ (3, n=1438)=5·4, P <0·001

(viii) Snakebite ever: Pearson X^2^ (6, n=1437)=20·7, P=0·002

*2. Mean variables (one-way ANOVA/Kruskal-Wallis test)*

(i) Age: F (3, 1434) = 43·6, P <0·001

(ii) Monthly income: F (3, 1410)= 14·0, P<0·001

(iii) Meat intake days in a month: F (3, 1433)= 35·2 P<0·001

(iv) Exposure to pesticides (years): Kruskal-Wallis [Chi-squared]= 3·3, P=0·34
