# Supplementary material for: A comparison of chronic kidney risk among returnee Nepalese migrant workers in the countries of the Gulf and Malaysia and non-migrants in Nepal: a population-based cross-sectional study
Source: BMC Nephrol. 2026 Mar 18;27:263. doi: 10.1186/s12882-026-04872-7 (PMC13122860; doi:10.1186/s12882-026-04872-7)
Supplement: Supplementary file 4 — Supplementary Material 4: Working conditions and lifestyle characteristics of male recent migrants (N=718) [file 12882_2026_4872_MOESM4_ESM.docx]

**Supplementary Table S4: Working conditions and lifestyle characteristics of male recent migrants (N=718)**

| **Variables** | **Number (%)** | **95% CI** |
| --- | --- | --- |
| **Rest episode(s) at work/day (n=717)** |  |  |
| One time or less | 365 (50.9) | 47.1 to 54.6 |
| Two times | 233 (32.5) | 29.1 to 36.1 |
| Three times | 89 (12.4) | 10.1 to 15.0 |
| Four times or more | 30 (4.2) | 2.8 to 5.9 |
| **Rest place at work** |  |  |
| Inside workplace | 272 (37.9) | 34.3 to 41.5 |
| Hostel or accommodation | 230 (19.2) | 28.6 to 35.6 |
| Shade | 138 (19.2) | 16.4 to 22.3 |
| Outside workplace | 39 (5.4) | 3.9 to 7.3 |
| Other (e.g., vehicle, canteen) | 39 (5.4) | 3.9 to 7.3 |
| **Regular day off once a week** |  |  |
| Always | 392 (54.6) | 50.9 to 58.3 |
| Sometimes | 244 (44.0) | 30.5 to 37.6 |
| Never | 82 (11.4) | 9.2 to 13.9 |
| **Physical level at work** |  |  |
| Heavy | 185 (25.8) | 22.6 to 29.1 |
| Moderate | 271 (37.7) | 34.2 to 41.4 |
| Light | 262 (36.5) | 32.9 to 40.1 |
| **Work setting** |  |  |
| Indoor | 332 (46.2) | 42.5 to 49.9 |
| Outdoor | 213 (29.7) | 26.3 to 33.1 |
| Indoor and outdoor mix | 173 (24.1) | 21.0 to 27.4 |
| **Heat exposure level at work** |  |  |
| High | 327 (45.5) | 41.8 to 49.3 |
| Moderate | 145 (20.2) | 17.3 to 23.3 |
| Low or none | 246 (34.3) | 30.8 to 37.9 |
| **Water availability at work** | 700 (97.5) | 96.1 to 98.5 |
| **Easy toilet access at work** | 697 (97.1) | 95.6 to 98.2 |
| **Dust exposure at work** |  |  |
| Always | 275 (38.3) | 34.8 to 41.9 |
| Sometimes | 120 (16.7) | 14.0 to 19.6 |
| Rarely or never | 323 (45.0) | 41.3 to 48.7 |
| **Chemical exposure at work** |  |  |
| Always | 69 (9.6) | 7.5 to 12.0 |
| Sometimes | 71 (9.9) | 7.8 to 12.3 |
| Rarely or never | 578 (80.5) | 77.4 to 83.3 |
| **Pesticide exposure at work** |  |  |
| Always or sometimes | 13 (1.8) | 0.9 to 3.1 |
| Never or rarely | 705 (98.2) | 96.9 to 99.0 |
| **Painkiller use** |  |  |
| Minimum once a week | 29 (4.0) | 2.7 to 5.7 |
| More than once a week | 22 (3.1) | 1.9 to 4.6 |
| Sometimes | 491 (68.4) | 64.8 to 71.8 |
| Never | 176 (24.5) | 21.4 to 27.8 |
| **Alcohol intake while abroad (yes)** | 384 (53.5) | 49.7 to 58.2 |
| **Frequency consuming one standard drink of alcohol (n=384)** |  |  |
| Daily | 7 (1.8) | 0.7 to 3.7 |
| 3-4 days a week | 18 (4.7) | 2.8 to 7.3 |
| 1-2 days a week | 134 (34.9) | 30.1 to 39.9 |
| 1-3 days a month | 148 (38.5) | 33.6 to 43.6 |
| Less than once a month | 77 (20.0) | 16.2 to 24.4 |
|  | **Mean (SD)** |  |
| Average working hours/day | 10.7 (0.1) | 10.5 to 10.8 |
| Average rest duration/day (minutes) | 76.7 (1.3) | 74.2 to 79.2 |
| Average no of days off in a month | 2.4 (0.1) | 2.2 to 2.5 |
