# Supplementary material for: A comparison of chronic kidney risk among returnee Nepalese migrant workers in the countries of the Gulf and Malaysia and non-migrants in Nepal: a population-based cross-sectional study
Source: BMC Nephrol. 2026 Mar 18;27:263. doi: 10.1186/s12882-026-04872-7 (PMC13122860; doi:10.1186/s12882-026-04872-7)
Supplement: Supplementary file 5 — Supplementary Material 5: Factors associated with reduced mean eGFR among male recent migrants adjusted for confounding factors, known risk factors and excluding occupational mediators [file 12882_2026_4872_MOESM5_ESM.docx]

**Supplementary Table S5: Factors associated with reduced mean eGFR among male recent migrants adjusted for confounding factors, known risk factors and excluding occupational mediators**

| **Variables** | **Estimate coefficient (95% CI)** | **P value** |
| --- | --- | --- |
| Migration duration (years) | 0.02 (-0.2 to 0.2) | 0.89 |
| Age (per decade older) | -7.4 (-8.8 to -5.9) | <0.001*** |
| Yearly household income (per 50,000 Nepalese Rupees higher) | 0.01 (-0.1 to 0.1) | 0.71 |
| Meat intake days (per month) | -0.03 (-0.1 to 0.1) | 0.58 |
| History of kidney disease (yes) | -8.9 (-19.8 to 2.1) | 0.10 |
| Hypertension or diabetes or on medication (yes) | -1.5 (-3.9 to 0.9) | 0.22 |
| Current or past drinker (yes) | -1.1 (-3.6 to 1.3) | 0.37 |
| Current or past smoker (yes) | -2.3 (-4.2 to -0.3) | 0.02* |
| BMI (per kg/m^2^ higher) | -0.2 (-0.5 to -0.003) | 0.04* |
| **Occupation (ref: other^#^)** |  |  |
| Construction work | -2.5 (-5.1 to 0.1) | 0.06 |
| Factory work | -1.4 (-4.0 to 1.3) | 0.32 |
| Driving | -1.3 (-4.7 to 2.1) | 0.44 |
| Security guard | -8.8 (-14.6 to -3.1) | 0.003** |
| **Completed education**  (ref: Illiterate or informal education) |  |  |
| Primary | 0.2 (-2.8 to 3.1) | 0.91 |
| Secondary | -0.8 (-3.6 to 1.9) | 0.54 |
| Higher secondary or above | -3.5 (-9.9 to 2.8) | 0.27 |
| **Ethnicity (ref: Terai other)** |  |  |
| Terai Janajati | -1.4 (-5.6 to 2.8) | 0.51 |
| Terai Dalit | -5.3 (-7.9 to -2.7) | <0.001*** |
| Terai Brahmin/Chhetri | 0.1 (-3.8 to 4.1) | 0.95 |
| Muslim | -3.1 (-6.1 to -0.1) | 0.04* |
| *P value: * (<0.05), ** (<0.01), *** (<0.001); Other^#^= mainly office work, sales, hotel work, cleaning* | | |
